# Phosphoglycerate dehydrogenase positively regulates the proliferation of chicken muscle cells

**DOI:** 10.1016/j.psj.2022.101805

**Published:** 2022-02-24

**Authors:** Han Wang, Moran Hu, Zhaoxue Ding, Xiaolong Zhou, Songbai Yang, Zhonghao Shen, Feifei Yan, Ayong Zhao

**Affiliations:** Key Laboratory of Applied Technology on Green-Eco Healthy Animal Husbandry of Zhejiang Province, College of Animal Science and Technology, College of Veterinary Medicine, Zhejiang A&F University, Zhejiang 311300, China

**Keywords:** chicken, skeletal muscle, cell proliferation, PHGDH, FoxM1

## Abstract

Phosphoglycerate dehydrogenase (**PHGDH**) is the rate-limiting enzyme in the serine synthesis pathway. However, the regulatory role of PHGDH in muscle development is unclear. We report that the expression of PHGDH increased significantly during proliferation of chicken skeletal muscle satellite cells. Knockdown of PHGDH by an siRNA suppressed myoblast proliferation, whereas overexpression of PHGDH enhanced muscle cell proliferation. Furthermore, PHGDH promoted the expression of Forkhead box protein M1 (**FoxM1**). Knockdown of FoxM1 by an siRNA attenuated the proliferation of chicken muscle cells, whereas its overexpression significantly promoted proliferation. Additionally, siRNA-PHGDH inhibited pcDNA3.1-FoxM1-induced FoxM1 expression in chicken muscle cells. Moreover, PHGDH inhibition overcame the stimulation by pcDNA3.1-FoxM1 of cell cycle-related gene expression. We propose that PHGDH accelerates chicken muscle cell proliferation by increasing FoxM1 expression.

## INTRODUCTION

Chicken is one of the most popular domestic animals for meat production. The growth rate of muscle is an important indicator of broiler meat performance (Petracci and Cavani, 2012). Most Chinese indigenous broiler breeds grow slowly, whereas commercial broiler breeds, which have been genetically selected, achieve high meat production efficiency (Sarsenbek et al., 2013). Therefore, the growth and development of chicken skeletal muscle is important for broiler genetic improvement.

Myogenesis is a complex biological process in which muscle progenitor cells proliferate, migrate, and fuse into polynucleated cells to form muscle tubes, which differentiate to form muscle by regulating multiple signaling pathways and factors (Perry and Rudnick, 2000). Skeletal-muscle satellite cells (**SMSCs**) belong to the group of muscle stem cells, which are important in muscle growth, repair, and maintenance after birth (Sacco et al., 2008; Harding et al., 2016). Numerous genes are important for the proliferation and differentiation of skeletal muscle satellite cells, such as paired box 7 (**PAX7**), myogenic factor 5 (**Myf5**), myogenic differentiation antigen (**MyoD**), myogenin (**MyoG**), and myogenic regulatory factor 4 (**MRF4**) (Yablonka-Reuveni and Rivera, 1994; Zammit, 2008). However, the regulation of muscle growth is complex, and exploring the mechanisms of skeletal muscle development would enable animal productivity to be increased.

PHGDH is the rate-limiting enzyme in the serine synthesis pathway, catalyzing the formation of 3-phosphohydroxypyruvate from phosphoglycerate. Other metabolic intermediates, such as serine, glycine, cysteine, and phospholipids, are produced via this pathway and are indispensable for protein synthesis and cell growth. PHGDH gene expression significantly promotes cancer-cell proliferation (Zhao et al., 2020). The biosynthetic pathway mediated by PHGDH plays a key role in the development of pig skeletal muscle (Brown et al., 2016). In PHGDH-knockout mice, genes related to muscle and cartilage development were significantly downregulated (Furuya et al., 2008). However, the role of PHGDH in chicken muscle growth and development remains to be further investigated. However, the role of PHGDH in chicken muscle growth and development is unclear. Muscle satellite cells would proliferate to satellite cell-derived myoblasts and differentiate to produce multinucleated myotubes when isolated and cultured in vitro (Konigsberg et al., 1975; Bischoff, 1986; Yablonka-Reuveni and Rivera, 1994). Therefore, SMSCs may be useful for investigating the role of PHGDH in myoblast proliferation.

In gliomas, PHGDH interacts with the N-terminus of FoxM1 to hinder its ubiquitination, thus causing degradation of the proteasome and promoting the proliferation of tumor cells (Liu et al., 2013). However, the interaction between PHGDH and FoxM1 in muscle cells needs to be further explored. We speculate that PHGDH regulates the proliferation of chicken skeletal muscle satellite cells by acting on the FoxM1 gene.

We hypothesized that PHGDH regulates the proliferation of chicken skeletal muscle cells by acting on FoxM1. We explored the role and regulation of PHGDH and FoxM1 in skeletal muscle satellite cells of broiler. The results provide an insight into the mechanisms of broiler muscle development.

## MATERIALS AND METHODS

### Experimental Animals and Tissues

Three 42-day-old Chinese indigenous broilers of similar weight and reared under the same feeding conditions were purchased from a farm (Hangzhou, China). Samples were collected as 3 replicates from 8 tissues of each broiler. After collection, the samples were rapidly frozen in liquid nitrogen for RNA extraction. Ten-day-old chicken embryos were hatched in our laboratory, and fertilized eggs were purchased from the same farm. All animal procedures were approved by the Ethics Committee for Animal Experiments of Zhejiang A&F University and were performed in accordance with the Guidelines for Animal Experimentation of Zhejiang A&F University (Hangzhou, China).

### Cell Culture

Chicken skeletal muscle satellite cells were those used in our prior studies (Wang et al., 2020). After sterilizing 10-day-old breeding eggs with alcohol, they were placed on an egg tray, gently broken, and the shell was removed using tweezers. Tweezers were next used to remove the chicken embryo and the leg muscle of the chicken embryo was isolated and transferred a new 10-cm cell petri dish. Muscle samples were washed 3 times in PBS (HyClone, Logan, UT) containing penicillin-streptomycin (Solarbio, Beijing, China), and the skin, blood vessels, fat, and connective tissue were removed. The muscle was crushed into meat paste and digested with 0.25% trypsin-EDTA (Thermo Fisher, Shanghai, China) at 37°C for 10 min. An equal amount of DMEM/F12 (HyClone, Logan, UT) containing 10% fetal bovine serum (**FBS**) (Thermo Fisher, Shanghai, China) was added to stop digestion. The suspension was filtered through a 70-μm mesh sieve and centrifuged at 1200 r/min for 8 min at room temperature. The supernatant was discarded, and the cells were resuspended in DMEM/F12 with 15% FBS, incubated for 60 min, and the supernatant transferred to a new 10-cm petri dish. This step was repeated twice. The SMSCs were cultured in growth medium (**GM**), DMEM (HyClone, Logan, UT) supplemented with 10% FBS and 1% penicillin-streptomycin (Gibco, NY), and incubated in 5% CO_2_ at 37°C.

### RNA Oligonucleotides and Plasmids Construction

RNA oligonucleotides, including the small interfering RNA (**siRNA**) and siRNA negative control (**NC**), were designed and synthesized by RiboBio (Guangzhou, China). The sequences are listed in Table 2. PcDNA 3.1 PHGDH expression vector. Primers were designed according to the NCBI Reference Sequence: XM_422226.7 to amplify the entire coding sequence (**CDS**) region of PHGDH and were digested with *Nhe*I and *Xho*I. The primer sequences were as follows:

5’-GCTAGCATGGCCTTTGCCAAGCTGCA-3’ and 5’-GAATTCTTACATTTTGATGCCTTCC-3’. The PCR product was cloned into the pcDNA-3.1 vector (Invitrogen, CA). The PcDNA 3.1 FoxM1 expression vector was designed and constructed by TsingKe (Hangzhou, China).

### Cell Transfection

Upon cells reaching 70 to 80% confluence, Lipofectamine 3000 (Invitrogen, Shanghai, China) was used for transfection following the manufacturer's protocol. The isolated cells were seeded on a 6-well plate at approximately 1  ×  10^5^ per well. When the cell density reached 50 to 60%, the cells were maintained in serum-free medium for transfection. The recommended siRNA or plasmid concentration was added to a centrifuge tube containing Opti-MEM and incubated at room temperature for 5 min. The recommended liposome concentration was added to another centrifuge tube containing Opti-MEM and incubated at room temperature for 5 min. The above 2 reagents were mixed gently and incubated at room temperature for 20 min. The mixture was then added to the cells in each well and gently shaken. The cells were next cultured in a 5% CO_2_ incubator at 37°C. After 4 to 6 h, the cells were replaced with GM and incubated in 5% CO_2_ at 37°C. Co-transfection was performed as described previously (Wang et al., 2018). The siRNA-PHGDH or NC and pcDNA3.1-FoxM1 or pcDNA3.1-Control plasmids were co-transfected into the cells at a level half that used for individual transfection.

### 5-Ethynyl-2’-Deoxyuridine (EdU) assay

Cells were seeded in 12-well plates. After transfection for 4 to 6 h, chicken SMSCs were cultured in fresh GM containing 10 mM 5-ethynyl-2’-deoxyuridine (**EdU**) for 24 h. The cells were fixed, permeabilized, and stained using the EdU Apollo567 Kit (RiboBio, Guangzhou, China) following the manufacturer's instructions. Three randomly selected interfaces of each treatment were observed using a fluorescence microscope (Olympus, Tokyo, Japan). The numbers of EdU-positive nuclei and total nuclei were determined.

### Flow Cytometry

Cells were seeded in 6-well plates. After 24 h of transfection, the cells were harvested, washed, and fixed in 500 μL of precooled 70% ethanol at 4°C overnight. The cells were next washed twice with PBS and added to 100 μL of RNase A solution. Thereafter, 50 mg/mL PI solution (Solarbio, Beijing, China) was added to the cells and incubated for 30 min at 4°C away from light. A BD flow cytometer (Becton Dickinson, NJ) was used to evaluate the samples. Data were analyzed using ModFit software (Verity Software House, Topsham, ME).

### Immunofluorescence Staining

Cells at 70 to 80% confluence were used for immunofluorescence analysis. Cells grown in 12-well plates were washed three times with precooled PBS for 5 min and fixed with 4% paraformaldehyde for 15 min. The cells were next permeabilized in the wells with 0.25% Triton X-100 for 10 min and blocked at 4°C overnight. Thereafter, cells were incubated with anti-Pax7 primary antibody (Abcam, Shanghai, China) for 1 h at room temperature. Subsequently, a fluorescent secondary antibody (Thermo Fisher, Shanghai, China) was incubated with the cells for 1 h at room temperature. 4’,6-Diamidino-2-phenylindole (DAPI) (Invitrogen, CA, USA) was added to stain nuclei and cells were incubated for 15 min at room temperature. A fluorescence microscope (Olympus) was used to observe the samples.

### Quantitative real-time RT-PCR

The TRIzol method was used to extract total RNA. Total RNA from tissues or cells was obtained using RNAiso reagent and treated with DNase I (TaKaRa, Kyoto, Japan). The concentration and purity of RNA were determined using an Agilent Bioanalyzer 2100 spectrophotometer (Agilent Technologies, CA). cDNA synthesis was performed using the 5X All-In-One RT MasterMix Transcription Kit (abm, Zhenjiang, China) according to the manufacturer's instructions, and cDNA was stored at –20°C for later use. mRNA amounts were determined by RT-PCR using a CFX96 instrument (Bio-Rad, CA) with Eva Green 2x qPCR Master Mix (abm). The internal control was GAPDH. Differential expression analysis was performed by the 2^−ΔΔCt^ method (Livak and Schmittgen, 2001). Primer sequences are listed in Table 1.

**Table 1:** Primers used in the real-time PCR.

Gene name	Sequence (5′-3′)	Product size
*GAPDH, PHGDH*	F: CGATCTGAACTACATGGTTTAC, R: TCTGCCCATTTGATGTTGC, F: AGCCAAAGCATCGGAGACA	153 bp, 151 bp
	R: AGCGAGGTCAGACAGTGGG	
*cyclin D1*	F: CACTTGGATGCTGGAGGT	110 bp
	R: GGCTTTTCTTGAGGGGTT	
*cyclin E*	F: AAAAGCAATACGAAAACC	302 bp
	R: AACCTCCATTAGCCAGTC	
*CDKN2A*	F: GGCCTCTGTCCTTCTCGCT	100 bp
	R: CTCAGAACCCGGCGCAGAAT	
*CDKN2B*	F: CGGATGAACTAGCCAACGCC	120 bp
	R: TCATCACCTGGATGGGGGTC	
*FoxM1*	F: ACTATCACAGCACCTTCCCT	240 bp
	R: TCAGTTCTCATCTTCCCCAG	

GAPDH: glyceraldehyde-3-phosphate dehydrogenase; PHGDH: Phosphoglycerate dehydrogenase; CDKN2A: cyclin dependent kinase inhibitor 2A; CDKN2B: cyclin dependent kinase inhibitor 2B; FoxM1: forkhead box M1.

**Table 2:** Sequence of RNA oligonucleotides.

Name	Forward (5’-3’)		Reverse (5’-3’)
RNA oligonucleotides			
Negative control	UUCUCCGAACGUGUCACGU	ACGUGACACGUUCGGAGAA	
siRNA- PHGDH	AGAUCGCCAUGCAGAUAGU	AGAUCGCCAUGCAGAUAGU	
siRNA- FoxM1	ACUCCUACAUGGCCAUGAU	AUCAUGGCCAUGUAGGAGU	

### Western Blot Analysis

Total cellular proteins were prepared and treated as described previously (Wang et al., 2017). Radio immunoprecipitation lysis buffer (Beyotime, Shanghai, China) with 1% phenylmethylsulfonyl fluoride (Beyotime, Shanghai, China) was used to extract total proteins from the cells. Proteins were separated by 12% SDS-PAGE and transferred to polyvinylidene fluoride membranes (Millipore, MA). After blocking with 5% BSA, the membranes were incubated with primary antibodies for PHGDH (HPA021241; Sigma, MO) and β-actin (6008-1-Ig; Proteintech, Wuhan, China). Thereafter, an HRP-labeled anti-rabbit/mouse IgG (Beyotime, Shanghai, China) was added as the secondary antibody. The blots were visualized using ECL reagent (Thermo Scientific, MA) and exposed to a chemiluminescence detection system (Tanon, Shanghai, China). Image J software was used for data analysis.

### Statistical Analysis

Data are means ± standard error of the mean (SEM), and each treatment was repeated at least three times. The unpaired Student's *t*-test was used to calculate *P*-values in SPSS 20.0 software (2010, SPSS Inc., Chicago, IL). *P* < 0.05 was considered indicative of significance and *P* < 0.01 of high significance.

## RESULTS

### Morphological Observation and Identification of Chicken Skeletal Muscle Satellite Cells

**Figure 1:**
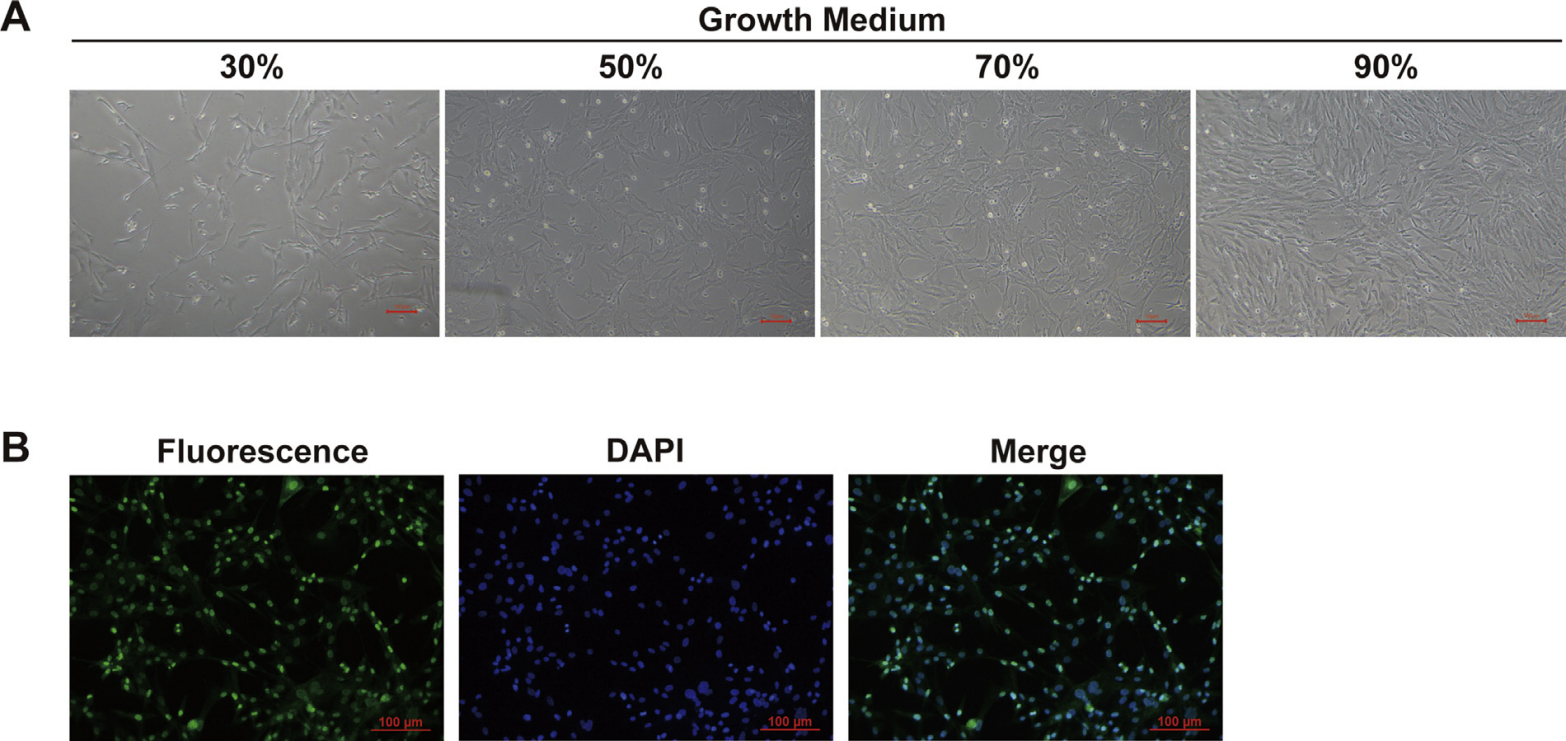
Morphological observation and immunofluorescence identification of chicken skeletal muscle satellite cells, (A) Morphological images of chicken SMSCs at 30%, 50%, 70%, and 90% confluence in growth medium (GM). (B) Pax7 was detected by immunofluorescence. DAPI was used to stain nuclei; a merged fluorescence and DAPI image is also shown.

Chicken skeletal muscle satellite cells are spherical before adherence. After 12 h, the cells began to adhere to the plate and became spindle-shaped at 30% confluence (Figure 1A). Subsequently, the cells began to proliferate and reached 50%, 70%, and 90% confluence (Figure 1A). Pax7 was positive in the nucleus (Figure 1B). Pax7, specifically expressed in quiescent and proliferating satellite cells, is important in SMSC regeneration and self-renewal (Seale et al., 2000; Halevy et al., 2004). Thus, Pax7 is a marker for satellite cell-derived myoblasts during cell proliferation, indicating that the isolated cells were SMSCs.

### PHGDH is Differentially Expressed in Tissues and Upregulated in Myoblast Proliferation

**Figure 2:**
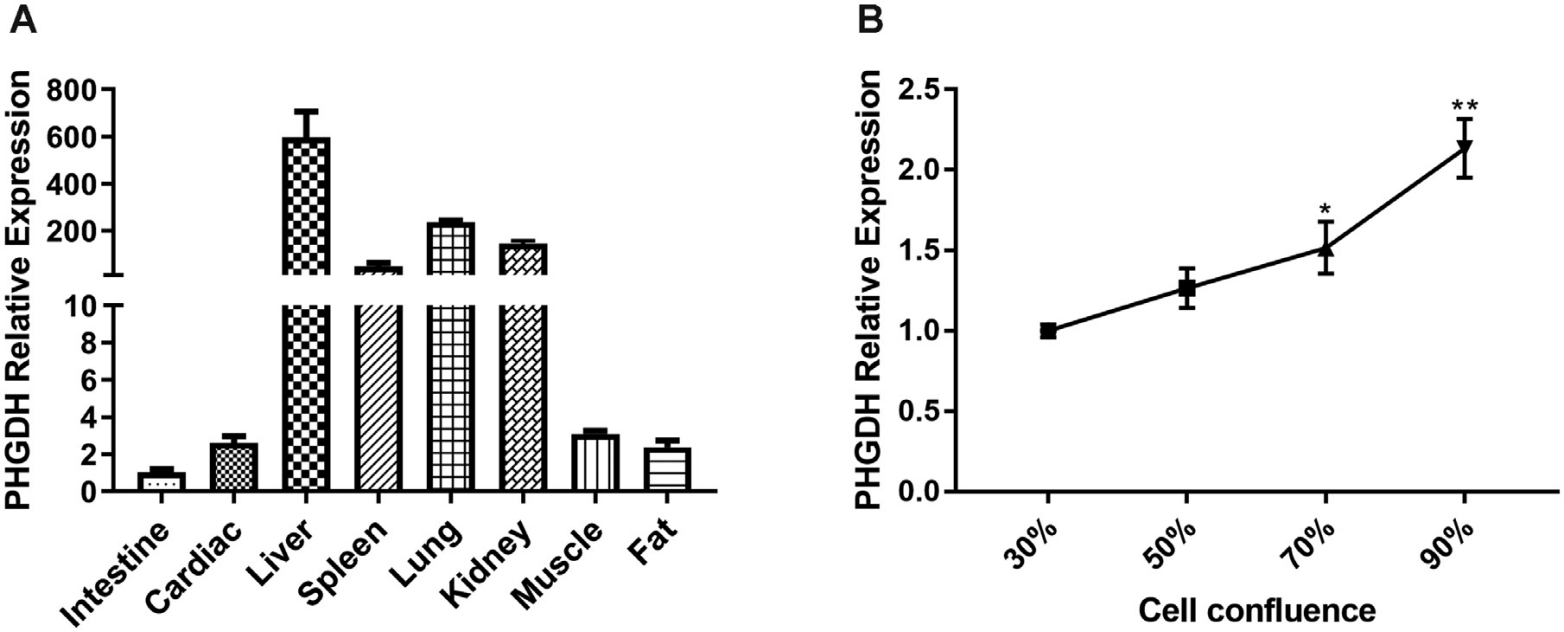
Expression of PHGDH in tissues and during chicken SMSC proliferation., (A) Expression of PHGDH in mouse intestine (control), cardiac, liver, spleen, lung, kidney, muscle, and fat. (B) Expression of PHGDH during proliferation. Fold changes are relative to 30% cell confluence. Data are means ± SEM of at least three independent experiments (* *P* < 0.05; ** *P* < 0.01).

To explore whether PHGDH participates in the proliferation of chicken myoblasts, we first determined its expression profile. PHGDH expression was high in liver, spleen, lung, kidney, muscle, fat, and cardiac tissue (Figure 2A). Furthermore, the PHGDH mRNA level increased progressively during proliferation (Figure 2B). Therefore, PHGDH is implicated in chicken skeletal myogenesis.

### Knockdown of PHGDH Attenuates Chicken Myogenesis

**Figure 3:**
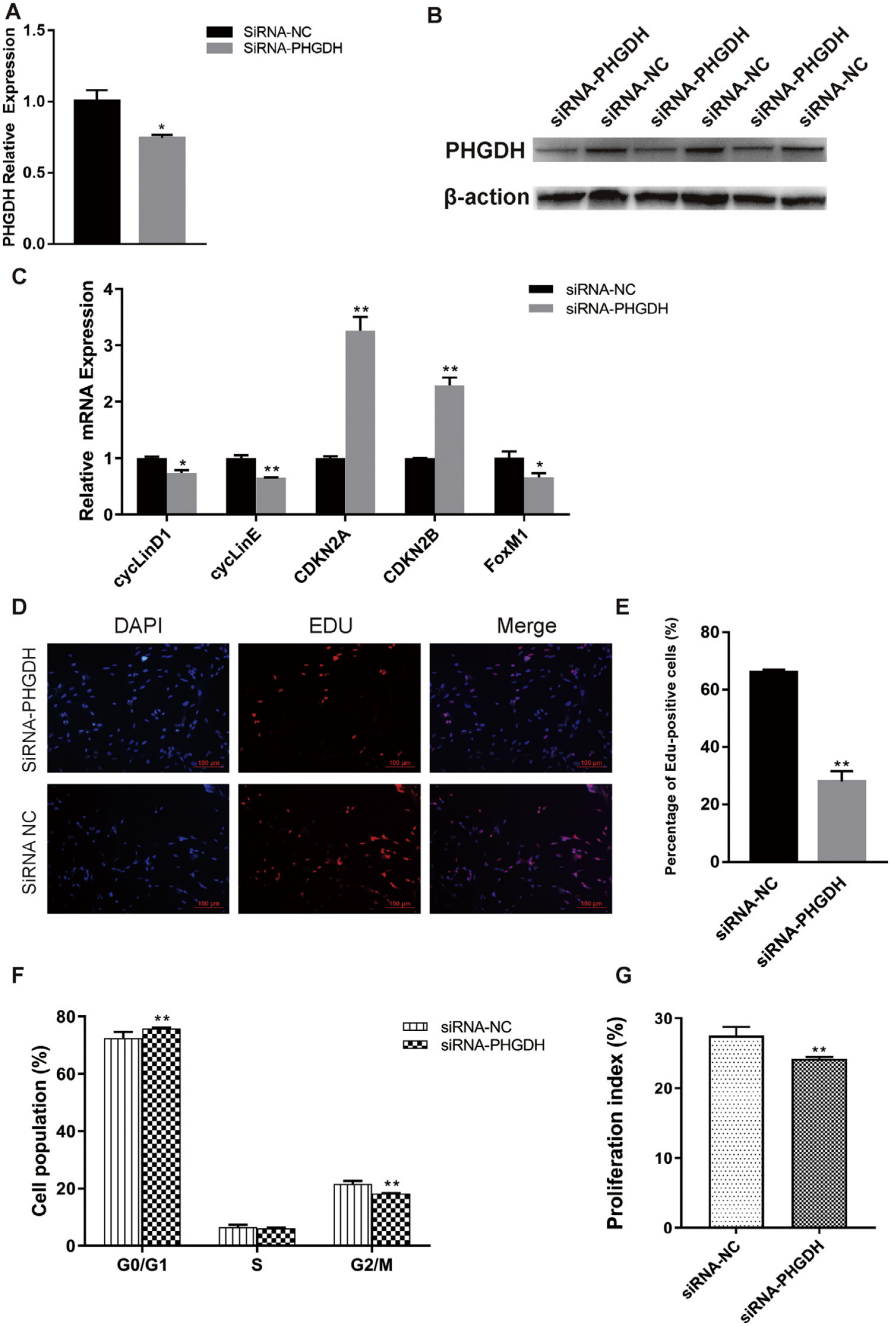
Downregulation of PHGDH inhibits chicken myoblast proliferation., (A) PHGDH mRNA level at 24 h post-transfection in GM. (B) PHGDH protein level at 24 h post-transfection in GM. (C) Expression of FoxM1 and cell cycle-related genes at 24 h post-transfection. (D) After transfection with siRNA-PHGDH or NC for 24 h, cells were fixed for EdU staining (red). Scale bar, 100 μm. (E) Proportion of EdU-positive cells. (F) Cell cycle distribution by PI flow cytometry. (G) Proliferation index was calculated as [(S+G2/M)/G0/1+S+G2/M)] × 100%. Data are means ± SEM of at least three independent experiments (* *P* < 0.05; ** *P* < 0.01).

To evaluate the role of PHGDH in chicken myoblast proliferation, synthetic siRNA or NC was transfected into chicken SMSCs to knockdown the expression of PHGDH (Figure 3A and B). The anti PHGDH siRNA suppressed the expression of FoxM1 and 2 cell-cycle activators, cyclin D1 and cyclin E (Figure 3C). Conversely, the mRNA levels of two cyclin-dependent kinase inhibitors, CDKN2A and CDKN2B, were significantly upregulated (Figure 3C). Also, the proportion of EdU-positive cells was reduced by inhibition of PHGDH (Figure 3D and E). In addition, PHGDH interference blocked the number of cells in the G0/G1 period, accompanied by a significant reduction in the cell proliferation index (Figure 3F and G). Therefore, PHGDH knockdown inhibited myogenesis.

### Upregulation of PHGDH Enhances Chicken Myogenesis

**Figure 4:**
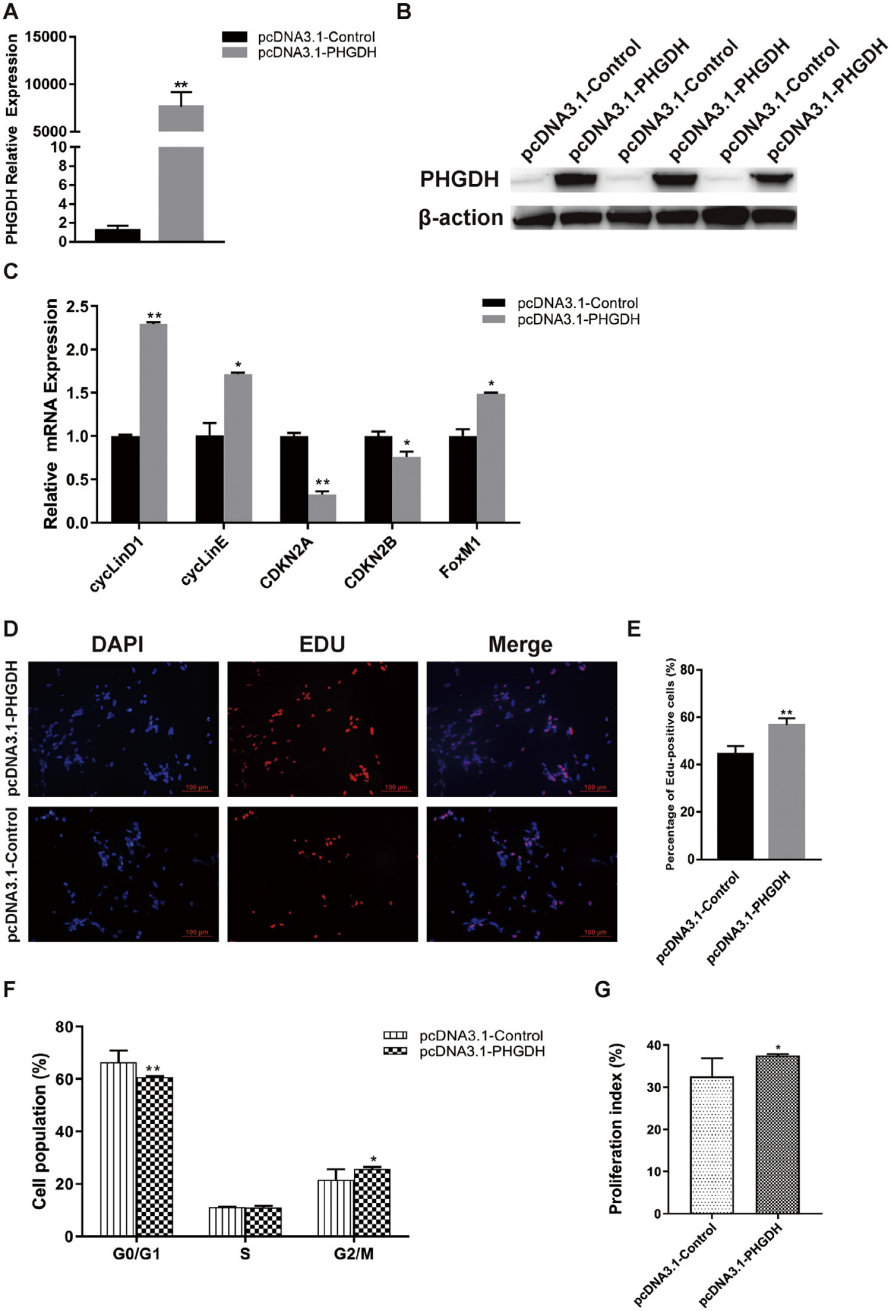
Plasmid-mediated upregulation of PHGDH promotes chicken myoblast proliferation., (A) PHGDH mRNA level at 24 h post-transfection in GM. (B) PHGDH protein level at 24 h post-transfection in GM. (C) Expression of FoxM1 and cell cycle-related genes at 24 h post-transfection. (D) After transfection with pcDNA3.1-PHGDH or pcDNA3.1-Control for 24 h, cells were fixed for EdU staining (red). Scale bar, 100 μm. (E) Proportion of EdU-positive cells. (F) Cell cycle distribution by PI flow cytometry. (G) Proliferation index was calculated as [(S+G2/M)/G0/1+S+G2/M)] × 100%. Data are means ± SEM of at least 3 independent experiments (*P < 0.05; **P < 0.01).

To confirm a role for PHGDH in attenuating myoblast proliferation, PHGDH overexpression cells were generated using pcDNA 3.1-PHGDH. The expression of PHGDH was increased after transfection in chicken cells (Figure 4A and B). The mRNA levels of FoxM1, cyclin D1, and cyclin E increased significantly, whereas those of CDKN2A and CDKN2B decreased (Figure 4C). The pcDNA 3.1-PHGDH group had a higher percentage of proliferating cells than the control group according to EdU assay (Figure 4D and E). The proportion of G1 phase cells was markedly lower than the control group, whereas the proportion of cells in G2/M phase and the proliferation index were significantly higher than the control group in PHGDH overexpression cells (Figure 4F and G). In summary, overexpression of PHGDH promoted the proliferation of chicken myoblasts.

### FoxM1 has a Positive Effect on Myogenic Proliferation

**Figure 5:**
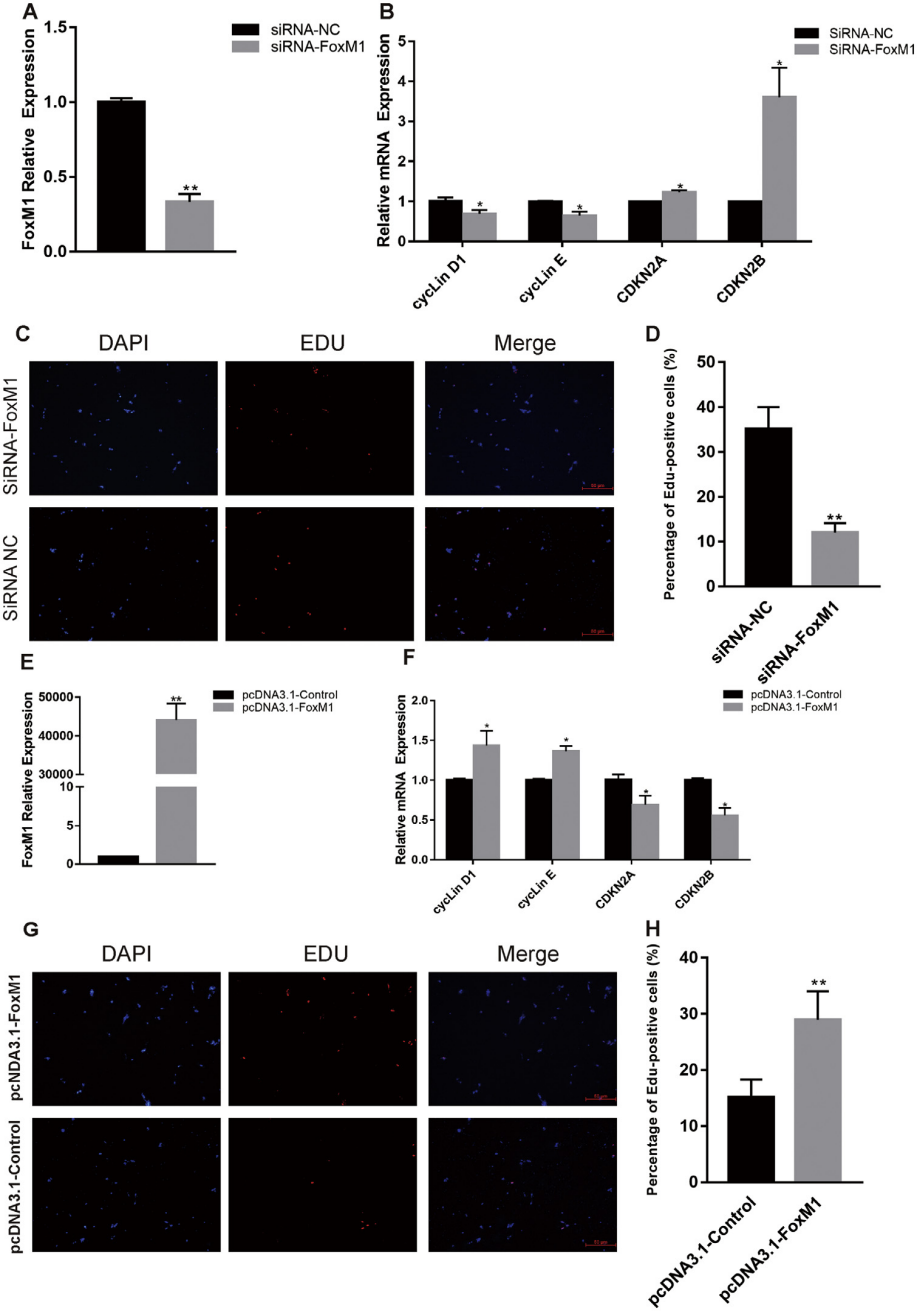
Effect of FoxM1 on proliferation of chicken muscle cells., (A) FoxM1 mRNA level at 24 h post-transfection in GM. (B) Expression of cell cycle-related genes at 24 h post-transfection. (C) After transfection with siRNA-FoxM1 or siRNA-NC for 24 h, cells were fixed for EdU staining (red). Scale bar, 100 μm. (D) Proportion of EdU-positive cells. (E) FoxM1 mRNA level at 24 h post-transfection in GM. (F) Expression of cell cycle-related genes at 24 h post-transfection. (G) After transfection with pcDNA3.1-FoxM1 or pcDNA3.1-Control for 24 h, cells were fixed for EdU staining (red). Scale bar, 100 μm. (H) Proportion of EdU-positive cells. Data are means ± SEM of at least three independent experiments (* *P* < 0.05; ** *P* < 0.01).

To determine whether FoxM1 influences myoblast proliferation, we used FoxM1 siRNA and pcDNA 3.1 vector to inhibit and promote the expression of FoxM1, respectively. Transfection of FoxM1 siRNA or FoxM1 pcDNA3.1 into chicken myoblasts decreased or increased the mRNA level of FoxM1, respectively (Figure 5A and E). As shown in Figure 5B, the mRNA levels of cyclin D1 and cyclin E were decreased significantly by inhibition of FoxM1 expression, but the expression of CDKN2A and CDKN2B was promoted. However, FoxM1 overexpression exerted the opposite effects on the mRNA levels (Figure 5F). EdU staining showed that the siRNA-treated group had a lower proportion of proliferating cells than the NC group, whereas the overexpression group had more EdU-positive cells than the control group (Figure 5C, D, G and H). Therefore, FoxM1 promotes the proliferation of chicken myoblasts.

### FoxM1 Expression is Downregulated by PHGDH

**Figure 6:**
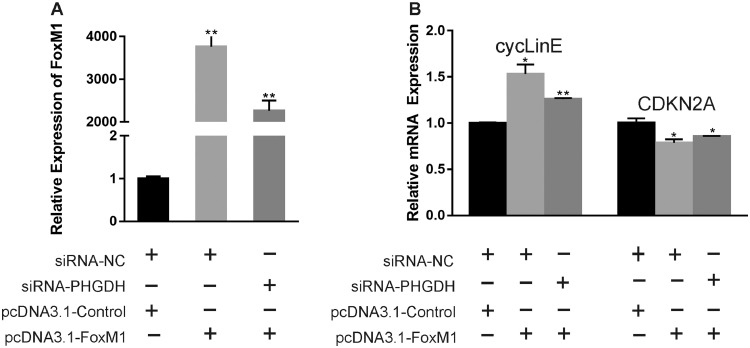
Effect of PHGDH on FoxM1 expression in chicken muscle cells., (A) FoxM1 mRNA level at 24 h post-transfection in GM. (B) Expression of cell cycle-related genes at 24 h post-transfection. Data are means ± SEM of at least three independent experiments (* *P* < 0.05; ** *P* < 0.01).

We next interfered with PHGDH expression in cells treated with pcDNA3.1-FoxM1. The siRNA-PHGDH inhibited pcDNA3.1-FoxM1-induced FoxM1 expression in chicken muscle cells (Figure 6A). Moreover, PHGDH inhibition suppressed the stimulation by pcDNA3.1-FoxM1 of the expression of cell cycle-related genes (Figure 6B). Therefore, PHGDH may influence chicken myoblast proliferation by regulating the expression of FoxM1.

## DISCUSSION

PHGDH is upregulated in multiple tumor types, inhibiting tumor-cell proliferation, suggesting utility as a therapeutic target for cancer (Li et al., 2021). PHGDH is expressed in multiple tissues, including skeletal muscle. PHGDH is implicated in pig and mouse skeletal muscle development (Brown et al., 2016; Brearley et al., 2019). However, no study has investigated its function in skeletal myoblast proliferation, especially in broiler chickens.

In this study, the expression of PHGDH increased during chicken muscle cell proliferation, indicating that PHGDH may promote skeletal muscle myogenesis. PHGDH catalyzes the first step in the synthesis of serine, a necessary precursor for the synthesis of various cell proliferation-related biomolecules (Yoshida et al., 2004). PHGDH significantly increased the expression of FoxM1 during chicken myoblast proliferation. PHGDH reportedly modulates FoxM1 expression, thus promoting the expression of the cell proliferation marker gene cyclin D1 and the proliferation of tumor cells (Liu et al., 2013). PHGDH inhibition or overexpression down and upregulated cell-cycle regulatory protein genes (cyclin D1 and cyclin E), whereas 2 cyclin-dependent kinase inhibitor genes (Cdk2A and Cdk2B) showed the opposite expression trend.

Knockdown of PHGDH suppressed proliferation of chicken myoblasts and resulted in their accumulation at the G1 phase. The cell cycle is regulated by cyclin and the corresponding cyclin-dependent kinase (CDK). Decreased expression of cyclin D1 and cyclin E represses cell proliferation by arresting muscle cells at the G0/G1 stage (Wu et al., 2015; Tsai et al., 2017). Also, increased expression of CDKN2A and CDKN2B arrested cells at the G0/G1 stage (Iolascon et al., 1996).

The loss of FoxM1 in muscle satellite cells induces muscle atrophy by reducing cyclin gene expression in mice (Chen et al., 2020). In this study, FoxM1 upregulated the expression of cyclin D1 and cyclin E in chicken muscle cells, downregulated the expression of CDKN2A and CDKN2B, and increased the proliferation index.

Furthermore, PHGDH partially reversed the pcDNA3.1-FoxM1-induced FoxM1 and cell cycle-related gene expression. Therefore, PHGDH might affect the expression of cell cycle-related proteins by acting on the FoxM1 gene, thus regulating the proliferation of chicken skeletal muscle satellite cells. However, we explored the effect of PHGDH on FoxM1 only at the mRNA level. Previous studies have found that the PHGDH-mediated serine synthesis pathway promotes MYC expression in lymphoma cells (Białopiotrowicz et al., 2020). MYC binds to the promoter region of FoxM1 in Myc overexpression cells, significantly increasing FoxM1 expression (Pan et al., 2018). Therefore, PHGDH may regulate the transcription of FOXM1 in muscle via transcription factors, such as MYC. Further studies are needed to verify the interaction between PHGDH and FoxM1 in chicken skeletal muscle.

We have reported that the daily weight gain of floor-reared broilers is higher than that of cage-reared broilers under the same feeding conditions, and that PHGDH expression is higher in the leg muscles of floor-reared broilers. Based on our current results, we speculate that PHGDH may affect the muscle development of broilers under different feeding modes. Taken together, our findings suggest a novel mechanism by which PHGDH promotes chicken skeletal muscle cell proliferation.
